# Epidemiology of Injuries Treated Among Male Youth Football Players During the 2021 Top Cup in Cameroon

**DOI:** 10.7759/cureus.94644

**Published:** 2025-10-15

**Authors:** Maurice Douryang, Leonard Tanko Tankeng, Hyacinte Trésor Ghassi, Kelly Jane Tsafack Nanfosso, Ayrton Moiroux-Sahraoui, Florian Forelli

**Affiliations:** 1 Department of Physiotherapy and Physical Medicine, University of Dschang, Dschang, CMR; 2 Department of Surgery and Surgical Specialties, University of Douala, Douala, CMR; 3 Department of Physical Medicine and Rehabilitation, Evangelical University Institute of Cameroon, Bandjoun, CMR; 4 Department of Orthopaedic and Trauma Surgery, Université Félix Houphouët-Boigny, Abidjan, CIV; 5 Department of Orthopaedic Surgery, Clinic of Domont, Domont, FRA; 6 Department of Sports Rehabilitation, Orthosport Center, Domont, FRA; 7 Department of Physical Medicine and Rehabilitation, Haute Ecole Spécialisée de Suisse Occidentale (HES-SO) University of Applied Sciences and Arts of Western Switzerland, Delémont, CHE

**Keywords:** cameroon, global epidemiology, sport injury, youth athlete, youth soccer

## Abstract

Objective

To investigate the epidemiological profile of injuries sustained during the 2021 Top Cup in Dschang, Cameroon, in order to inform prevention strategies and rehabilitation measures.

Methods

In this retrospective epidemiological study, we recorded the daily number of injury occurrences among male youth football athletes based on the medical staff reports from the 2021 Top Cup held in Dschang, Cameroon.

Results

A total of 128 male players were expected to participate in the tournament; however, only 117 players were present and sustained at least one injury. The mean age of the players was 11.9 ± 1.9 years. Most participants were enrolled in secondary school (93.16%). The overall injury incidence was 279.4 injuries per 1,000 player-hours (95% CI: 229.2-330.6). Muscle contusions were the most frequent injury type, accounting for 78 (66.7%) cases, followed by lacerations, 28 (23.9%). Muscle/tendon injuries represented 85 (72.6%) of all injuries. Most injuries were minor, 112 (95.7%), and the majority resulted from contact, 103 (88.0%). The lower limb was the most affected region, 82 (70.1%), particularly the knee, foot/ankle, and leg. Treatment was predominantly conservative, with only one case requiring surgery for a tibial fracture.

Conclusion

This study highlights a high incidence of football-related injuries among male players during a youth tournament, with most injuries being minor, contact-related, and affecting the lower limbs. Muscle contusions were the predominant injury type. These findings underscore the need for targeted injury prevention strategies, improved playing conditions, and access to medical supervision in youth sports settings to enhance player safety and support early rehabilitation.

## Introduction

Football, also known as soccer, is the most popular youth sport globally and holds a particularly significant cultural and recreational role in African countries, including Cameroon [1]. With increasing participation among children and adolescents, football has become a vital platform for both physical development and talent identification [1,2]. In Africa, tournaments such as the Top Cup serve as pivotal events for nurturing young talent. The Top Cup, established in 1982 by “Les Brasseries du Cameroun,” provides youth players with opportunities to train in professional environments and a pathway toward a football career. However, these tournaments also expose young players to injury risks, which could jeopardize their aspirations.

Youth football players in Africa, much like their counterparts globally, face similar injury risks. Studies on youth football injuries report incidence rates varying from 2.0 to 19.4 injuries per 1,000 hours of play [3-5]. These injuries are more frequent during competitive matches than training sessions due to the increased intensity and physical demands [6]. Similar findings have been documented in international studies, such as Mandorino M et al., which offers a detailed analysis of injury incidence and risk factors among youth soccer players worldwide [7]. Their analysis reveals that lower limb injuries, especially those affecting the knee and ankle, are the most frequent, often resulting from player-to-player collisions, sudden changes in direction, or overuse [8,9]. Additionally, the review emphasizes the variability of injury incidence based on age, playing surface, and competitive level, factors that may also apply to youth players in Africa. These insights further reinforce the need for region-specific studies, such as the current investigation, to address the unique conditions of youth football in Africa.

Despite the growing popularity of football in Africa, limited data are available on youth football injuries in the region. However, studies from African countries highlight the importance of understanding injury patterns specific to the continent. For instance, Owoeye OB et al. investigated injuries among Nigerian youth football players and reported an injury incidence rate of 7.2 injuries per 1,000 hours, with a predominance of lower limb injuries [10]. Similarly, studies in South Africa have shown that most injuries occur during matches, with the knee and ankle being the most commonly affected areas [11-13]. These findings are consistent with global trends but underscore the need for more region-specific data to inform prevention strategies tailored to African contexts.

Although injury trends in African youth football remain underreported, such gaps highlight the importance of research efforts like this study. Injuries among youth athletes in the Top Cup have not been systematically studied, presenting an opportunity to contribute valuable epidemiological data for this population. By situating these findings within the broader context of African youth football, this study aims to offer insights not only for Cameroon but also for youth football programs across the continent.

This study investigates the epidemiological profile of injuries sustained during the 2021 Top Cup in Dschang, Cameroon, in order to inform prevention strategies and rehabilitation measures relevant to Cameroonian and African stakeholders, as well as the Sports and Exercise Medicine community, to promote safer participation of youth football players in these career-defining tournaments. To the best of our knowledge, this is the first study conducted in Cameroon, and more broadly in Africa, that specifically documents injury patterns among male youth football players during a youth tournament, thereby filling a critical gap in regional sports medicine literature.

## Materials and methods

Study design

This study was a retrospective, descriptive epidemiological investigation conducted to document musculoskeletal injuries sustained during a youth football tournament, the 2021 edition of the Top Cup, held in Dschang, in the West Region of Cameroon. The tournament took place over a 16-day period, from June 24 to July 10, 2021.

Study setting

The tournament was held on a community football field located in the city of Dschang. The playing surface consisted of dry, compacted soil without natural or artificial grass. The field was irregular and lacked standard markings or protective infrastructure. Adapted goalposts were used, and no official measurement of the field’s dimensions was conducted. All matches were played under informal conditions typical of grassroots youth tournaments in the region, and no standardized safety or training protocols were in place for the participants.

Study population

The study population comprised all 128 male players (all Cameroonian) who were officially registered and expected to participate in the national tournament. The players were non-professional, casual athletes with no formal club affiliation or structured training background; they were selected informally to take part during the school holiday period across the 10 regions.

Inclusion criteria for analysis included: (1) male sex, (2) participation in at least one official match during the tournament, and (3) occurrence of a musculoskeletal injury that required medical attention and was documented by the medical team. Players who did not present with an injury or had incomplete or unverifiable injury documentation were excluded from the final analysis. A total of 117 injured players met the inclusion criteria, as they participated in the tournament.

Sampling method

An exhaustive sampling approach was used. All injury cases that met the inclusion criteria during the entire tournament period were included in the analysis. No sampling or selection was performed, as the study aimed to describe all documented injuries within the defined target population.

Injury definition

For the purpose of this study, an injury was defined as any physical damage sustained by a player during the tournament that required evaluation or treatment by a medical professional. This operational definition aligns with the recommendations from the FIFA Medical Assessment and Research Centre Consensus Statement on football injury surveillance [14].

Data collection and statistical analysis

For this study, data were collected from the tournament injury register after obtaining the appropriate authorization. Injuries were documented using standardized data extraction forms completed by the official medical team throughout the tournament. Each reported case was subsequently classified according to the criteria outlined in the International Olympic Committee (IOC) Consensus Statement on Injury Surveillance in Sports [15]. Only musculoskeletal injuries that required medical attention or resulted in time loss from football participation were included in the analysis. Each injury was assessed on-site by an experienced medical doctor or physiotherapist.

Data were entered, cleaned, and analyzed using Microsoft Excel 2016. The variables collected included injury incidence, type of injury, anatomical location (categorized as upper limb, lower limb, head/neck, or trunk), injury mechanism (contact vs. non-contact), and injury severity (minor, moderate, or severe). Categorical variables were summarized using frequencies and percentages, while continuous variables such as age were described using means, standard deviations, and ranges.

Injury incidence was estimated using the IOC-recommended formula: (number of injuries ÷ total exposure hours) × 1,000.

To approximate exposure, we reconstructed the tournament structure. Nine teams competed across two successive pool phases (three pools of three teams, followed by two pools of three teams), then semifinals and finals. Each team played an average of two to six matches depending on progression. A total of 19 matches were estimated, each lasting approximately one hour with 22 on-field players, resulting in a total estimated exposure of 418 player-hours. As this was a retrospective descriptive study, no inferential statistical tests were applied.

Ethical considerations and informed consent

Data handling followed ethical guidelines, respecting the principles of confidentiality and privacy regarding the medical and personal information of the injured players. Informed consent for all athletes included in this study was systematically obtained from their parents or legal guardians three months before the tournament, stating that data from the tournament could be used for research purposes. Assent was obtained from the children prior to data collection.

The study received ethical clearance from the Institutional Ethics Committee for Research on Human Health of the University of Douala, Douala, Cameroon (No468IEC-UD/10/2024/M).

## Results

Descriptive profile of players

A total of 128 male players were officially registered for the tournament, among whom 117 sustained at least one injury and were included in the analysis, as they attended the football event. The ages of the injured players ranged from 10 to 12 years, with a mean age of 11.9 ± 1.9 years. Regarding educational level, the majority of injured players were enrolled in secondary school, accounting for 93.16% of the sample, while 6.84% were in primary school. The injured players were distributed across the nine teams that took part in the tournament.

Injury incidence

A total of 117 players sustained injuries during the tournament. Based on an estimated 418 player-hours of exposure, the injury incidence rate was approximately 279.4 injuries per 1,000 player-hours of exposure (95% CI: 229.2-330.6) (Table 1).

**Table 1:** Calculation of exposure and injury incidence.

Item	Value
Total number of matches played	19
Match duration	60 minutes (1 hour)
Number of players exposed per match	22
Total exposure time (player-hours)	19 matches × 22 players = 418
Total number of injuries	117
Injury incidence rate	(117 ÷ 418) × 1,000 = 279.4 injuries per 1,000 player-hours
95% CI of incidence rate	229.2-330.6

Types of injuries recorded

During the Top Cup, muscle/tendon injuries were the most frequent, representing 72.6% (85 cases) of all reported injuries. These were followed by superficial tissue/skin injuries at 23.9% (28 cases), ligament or joint capsule injuries at 2.6% (3 cases), and bone injuries at 0.9% (1 case).

Within the muscle/tendon category, muscle contusions predominated (78 cases; 66.7%), while muscle strains (3 cases; 2.6%) and tendinopathies (4 cases; 3.4%) were less frequent. Lacerations accounted for all superficial tissue injuries (28 cases; 23.9%). Ligament/joint capsule injuries comprised two joint sprains and one dislocation, whereas bone injuries were limited to a single fracture. Additionally, one case of concussion without loss of consciousness (based on the Glasgow Coma Scale) was recorded (Table 2).

**Table 2:** Classification of recorded injuries.

Injury Type	Frequency (n = 117)	Percentage (%)
Muscle/tendon (n = 85; 72.6%)		
Tendinopathy	4	3.4
Muscle strain	3	2.6
Muscle contusion	78	66.7
Superficial tissue/skin (n = 28; 23.9%)		
Laceration	28	23.9
Bone (n = 1; 0.9%)		
Fracture	1	0.9
Ligament/joint capsule (n = 3; 2.6%)		
Joint sprain	2	1.7
Dislocation	1	0.9
Total	117	100

Distribution of injuries according to severity, mechanism, location, and body region

Of the 117 injuries recorded, the majority (95.7%) were classified as minor, while only five cases (4.3%) were considered severe, including one fracture, two sprains, one muscle elongation, and one concussion. Although the study used a three-level classification system (minor, moderate, and severe), no moderate injuries were identified in the dataset.

Contact was the predominant mechanism of injury, accounting for 88.0% of cases, compared to 12.0% for non-contact injuries. The lower limb was the most frequently affected body region (70.1%) (Table 3), particularly the knee (25.6%), foot and/or ankle (23.1%), and leg (16.2%). Other common injury sites included the abdomen (10.3%), elbow and hand (6.8% each), and head (4.3%). Injuries to the upper limb, trunk, and head/neck were less common than those affecting the lower limb.

**Table 3:** Location of injuries by affected body regions.

Injured Location/Area	Frequency (n = 117)	Percentage (%)
Lower limb (n = 82; 70.1%)		
Knee	30	25.6
Foot	27	23.1
Lower leg	19	16.2
Ankle	3	2.6
Thigh	2	1.7
Hip	1	0.9
Trunk (n = 12; 10.3%)		
Abdomen	12	10.3
Upper limb (n = 18; 15.4%)		
Elbow	8	6.8
Hand	8	6.8
Wrist	1	0.9
Shoulder	1	0.9
Head and neck (n = 5; 4.3%)		
Head	5	4.3
Total	117	100

**Figure 1:**
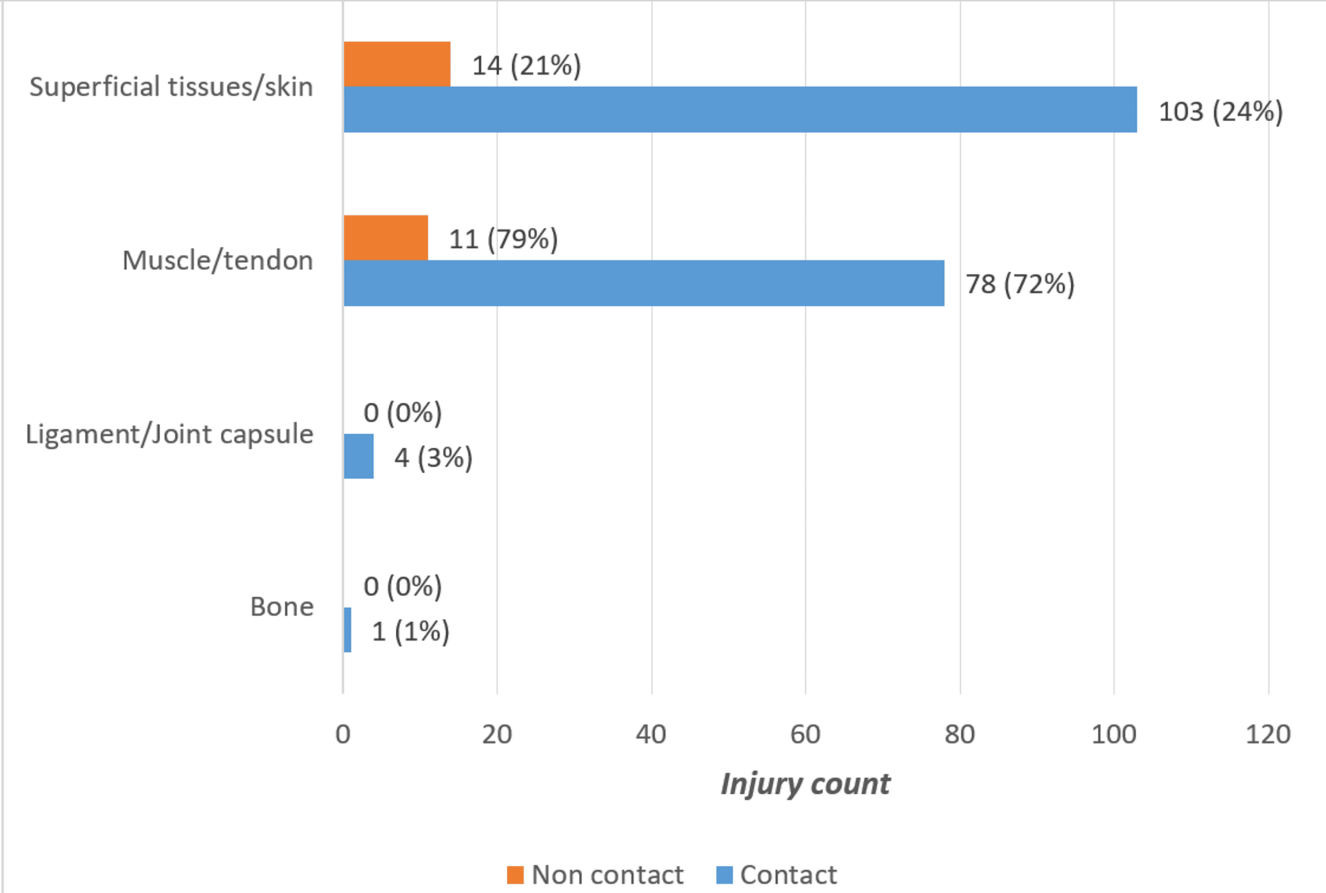
Distribution of causes in the affected tissues.

When cross-tabulating injury type and cause, muscle/tendon injuries emerged as the most frequent, occurring in both contact (72%) and non-contact (79%) cases (Figure 1). Superficial tissue injuries were also common, though predominantly contact-related, while ligament/joint capsule and bone injuries were rare and primarily caused by contact.

Treatment: conservative or surgical

Conservative treatment consisted of pain therapy, cryotherapy, casting or orthosis, and K-taping/strapping. In some cases, wound or skin injury treatment (dressing) was required. Conversely, surgical treatment was applied in a single athlete who sustained a fractured tibia.

## Discussion

The injury incidence rate observed in this study, 279.4 injuries per 1,000 player-hours of exposure, is notably higher than rates commonly reported in the literature on youth football, which typically range between 2.0 and 19.4 injuries per 1,000 hours in developed regions. This discrepancy may be attributed to differences in study design, injury definitions, exposure estimation, and the informal, non-professional status of players in our setting, as well as the high-intensity nature of the tournament assessed. For example, Junge A et al. reported an incidence of 6.0 injuries per 1,000 hours during youth tournaments, highlighting the role of match intensity in elevating injury risk [16]. Additionally, Pfirrmann D et al. noted that football is among the sports with the highest youth injury rates, especially during matches due to frequent physical contact [4]. The elevated incidence in our study may also be associated with contextual factors typical of informal youth competitions in Cameroon, such as limited use of protective equipment, uneven playing surfaces, inadequate coaching supervision, and minimal medical screening. These factors could potentially contribute to increased injury risk.

In terms of injury mechanisms, our findings are in line with those of Faude O et al., who conducted a systematic review showing that 60% to 90% of injuries in youth football are contact-based [6]. This high proportion reflects the physical nature of football, where tackles, headers, and frequent player-to-player interactions increase the risk of traumatic injuries [17]. This pattern is consistent across youth sports, as confirmed by Prieto-González P et al., who also reported a predominance of contact injuries in adolescent athletes, further emphasizing that youth football injury prevention must focus on techniques that reduce impact during play [6,18].

Our results also align with findings from Rössler R et al., regarding injury location, particularly the high incidence of lower limb injuries in youth football [18]. Rössler R et al. found that 76.3% of injuries occurred in the lower extremities, with the knee and ankle identified as the most vulnerable sites due to the demands of sprinting, jumping, and pivoting movements intrinsic to football [14]. This is corroborated by a study conducted by Robles-Palazón FJ et al., which found that lower extremity injuries accounted for nearly 80% of all injuries in youth football, with the knee, ankle, and foot being the most frequently affected areas [14,19].

However, discrepancies arise when examining the types of injuries. Our study found a high prevalence of muscle contusions and lacerations, while other studies, such as that by Rössler R et al., reported a significant proportion of ligament injuries, with joint sprains representing around 30.5% of total injuries [14]. These differences may reflect environmental factors, such as the type of playing surface. For instance, studies conducted on natural or artificial grass fields, as in Rössler R et al.’s work, often report more ligamentous injuries due to the stability and traction provided by these surfaces, whereas our study’s setting on sandy and rocky fields may increase the risk of falls and impact-related injuries to soft tissue rather than ligamentous structures [14].

In addition, the age and physical maturity of the players could influence injury patterns [20]. Light N et al. demonstrated that injury risk can vary significantly with player maturation, with younger, prepubescent athletes experiencing more acute, impact-related injuries due to lower body mass and strength, compared to older youth athletes who are more prone to ligamentous injuries as their muscle strength and playing intensity increase [21]. This underscores the importance of age-specific training and injury prevention strategies that accommodate the developmental stages of young athletes [20,22].

Finally, these findings highlight the need for tailored injury prevention strategies that take into account the specific injury profiles observed in youth football, as well as local factors such as playing surfaces and training intensity. Studies such as those by Owoeye OB et al. suggest that injury prevention in youth football can benefit from interventions incorporating strength and neuromuscular training, which can help reduce lower extremity injuries [10]. Furthermore, Kerr ZY et al. recommend the use of protective equipment, such as shin guards and high socks, and the promotion of safe playing techniques as effective strategies for mitigating injury risks in contact sports [10,23].

The results of this study suggest that playing surface significantly impacts the nature and frequency of injuries, particularly those affecting the lower extremities. Research consistently highlights that surface type, whether natural, artificial, or unregulated (e.g., sandy or rocky), plays a crucial role in determining the injury profile in youth sports. According to Thomson A et al., injuries tend to vary based on the stability and traction of the surface; grass and artificial turf, for instance, provide different traction forces that influence joint load and movement stability, which can directly affect the rate of ligament and joint injuries [24].

Studies comparing different surface types in youth sports, such as those by Ekstrand J et al., indicate that artificial and well-maintained natural surfaces tend to result in fewer acute injuries, especially non-contact injuries, compared to unregulated, uneven surfaces. In our context, the sandy and rocky playing fields may exacerbate the risk of falls and impact-related injuries, such as contusions and lacerations, due to their unpredictable and often irregular texture [25]. Ekstrand J et al. found that uneven surfaces increase the likelihood of falls and lower-limb injuries, as players have less stable footing, particularly during rapid changes in direction or speed [25].

Further supporting these findings, a study by Steffen K et al. on injury profiles associated with different surface types found that rough, unregulated fields led to a higher rate of soft-tissue injuries (e.g., contusions and abrasions) and lower-limb injuries due to the instability of footing. This aligns with our results, where muscle contusions and lacerations were prominent, possibly due to the direct contact of players with rough surfaces during falls [26].

Additionally, studies on youth soccer by Meyers MC highlight that less controlled environments, such as sandy or rocky fields, can lead to higher rates of joint sprains and ankle injuries [27]. Meyers demonstrated that uneven or soft surfaces, which fail to provide adequate support during high-impact movements, could contribute to excessive joint stress, increasing the risk of sprains and muscle strains. This suggests that players in our study likely experienced a higher incidence of these injury types due to the regional conditions of play [27].

Given these findings, enhancing the quality of playing surfaces could be a significant step in reducing injury risk for young athletes in this tournament context. Implementing safer, more stable surfaces or improving the condition of existing fields may mitigate injury risks associated with instability and surface irregularities. Such adaptations, coupled with appropriate protective equipment and training on safe movement patterns, could reduce the injury burden linked to the environmental factors identified in this study.

Given that the majority of injuries in this study were superficial contusions and contact-related, implementing specific prevention strategies could significantly reduce injury rates among young football players. Improving playing surface quality is one essential approach, as studies show that safe, stable surfaces can reduce the risk of traumatic injuries. Research by Soligard T et al. demonstrates that irregular or poor-quality surfaces contribute to increased fall-related injuries, such as contusions and abrasions. Upgrading to well-maintained grass or artificial turf could provide players with a safer environment, particularly in tournaments where intense physical interactions are common [28].

Protective equipment, especially reinforced shin guards, could further mitigate the impact of contact injuries. According to a study by Delaney JS et al., wearing high-quality shin guards is associated with reduced severity and incidence of lower-leg contusions and fractures. Protective equipment tailored for youth players, including shin guards with enhanced padding and coverage, is particularly valuable in preventing injuries from accidental kicks or tackles, which are frequent in youth football [29].

Education on safe playing techniques is another crucial component of injury prevention. Junge A and Dvorak J emphasize that educating young players on safe tackling and collision-avoidance techniques can reduce injury risk in contact sports [30]. Programs focused on proper body mechanics during tackles, falls, and evasive maneuvers can be particularly effective when introduced early in a player’s development. Furthermore, structured warm-up programs such as FIFA 11+ have been shown to reduce injury rates by promoting agility, strength, and balance, helping players better control their movements and avoid unnecessary contact [14,30].

Overall, implementing these strategies, improving playing surface quality, reinforcing protective equipment standards, and educating young players on safe techniques, could form a comprehensive approach to injury prevention. By adapting preventive measures to the context of youth football, tournament organizers and coaches can create safer playing conditions that support both player enjoyment and injury reduction.

The results of this study indicate a high prevalence of minor injuries, with most categorized as contusions or superficial injuries. This trend is consistent with findings in youth sports, where minor injuries are more common due to the lower intensity and shorter duration of play compared to adult leagues. However, continuous monitoring and early intervention are crucial in preventing the progression of minor injuries into more severe, long-term conditions. Studies suggest that without proper management, even minor injuries can escalate, particularly if players return to play too soon or without adequate recovery [31].

Implementing targeted injury prevention and recovery sessions after matches could be highly beneficial. A study by Hägglund M et al. found that structured recovery sessions, including cool-down exercises, stretching, and strength training, significantly reduce the risk of recurrent injuries [32]. This proactive approach can help young athletes heal fully from minor injuries, reducing the likelihood of recurrence and the potential progression into severe injuries requiring extended time away from play [32].

In addition, evidence supports the role of continuous injury surveillance as an effective strategy for identifying players at risk of injury progression. According to a systematic review by Collard DC et al., continuous monitoring of youth athletes’ injury patterns allows coaches and medical staff to intervene early, particularly important in contact sports like football, where cumulative effects from repeated minor injuries can lead to more severe musculoskeletal issues over time [33]. Surveillance programs that track injury occurrences, treatment, and recovery can help guide training adaptations and protective measures, such as limiting high-impact drills for recently injured players [33].

Moreover, prevention programs such as FIFA 11+ Kids, designed specifically for younger athletes, have shown success in reducing both minor and severe injuries through a combination of strength, balance, and coordination exercises [14]. Incorporating such programs into regular training can be instrumental in reducing the injury burden and allowing for better management of minor injuries before they progress to severe stages.

Thus, while minor injuries may appear less concerning in the short term, their cumulative impact and potential for escalation emphasize the need for structured prevention and recovery strategies. By focusing on consistent injury monitoring and targeted recovery sessions, teams can create safer sporting environments that prioritize both immediate and long-term athlete health.

Clinical and policy implementations

Policymakers in this tournament context may consider strategies such as distributing shin guards, ensuring play on natural pitches, and requiring long socks reaching knee level to reduce contact injuries and lacerations during play.

This study provides first-hand data for other researchers to pursue studies exploring factors associated with injuries in this population.

Limitations

As limitations of our study, the sample size was small, and the follow-up period was short, which may reduce the generalizability of our findings. In addition, missing variables such as BMI, player position, and experience level resulted in an incomplete picture of the study, which could be improved in future research. Investigating other factors (such as equipment use) associated with the occurrence of injuries would provide further insights for developing targeted policies to prevent injuries among young soccer athletes in Cameroon.

However, this study is the first in Central Africa to provide epidemiological data on injuries among young soccer athletes. Our findings will help guide larger-scale, long-term follow-ups to enable a better assessment of the current situation.

## Conclusions

The injury incidence during the 2021 Top Cup in Dschang was 279.4 injuries per 1,000 player-hours of exposure, with muscle contusions and lacerations being the most common injuries. The knee, foot and/or ankle, and leg were the most frequently affected body regions. These findings suggest that prevention strategies should focus on protecting the lower limbs and reducing contact-related injuries. Furthermore, ongoing research across multiple editions of the Top Cup should be encouraged to better understand the epidemiology and risk factors of youth football injuries in Cameroon. Such efforts would support the development of injury prevention measures tailored to the specific needs of this population.
